# The RioK1 network determines p53 activity at multiple levels

**DOI:** 10.1038/s41420-023-01704-7

**Published:** 2023-11-07

**Authors:** Michela Damizia, Gian Mario Moretta, Peter De Wulf

**Affiliations:** Department of Cellular, Computational, and Integrative Biology (CIBIO), University of Trento, 38123 Trento (TN), Italy

**Keywords:** Tumour-suppressor proteins, Cell signalling

## Abstract

By responding to a host of adverse conditions, ranging from DNA damage to viral infection, transcription factor p53 supports genomic stability, cellular health, and survival. Not surprisingly, tumours across the cancer spectrum carry mutations in p53, misexpress the protein, or dysregulate its activity. Several signalling pathways, many of which comprise oncogenic proteins, converge upon p53 to control its stability and activity. We here present the conserved kinase/ATPase RioK1 as an upstream factor that determines p53 activity at the DNA, RNA, and protein levels. It achieves this task by integrating the regulatory events that act on p53 into a coherent response circuit. We will also discuss how *RIOK1* overexpression represents an alternative mechanism for cancers to inactivate p53, and how targeting RioK1 could eradicate malignancies that are driven by a dysregulated RioK1-p53 network.

## Facts

- The conserved kinase/ATPase RioK1 promotes cell growth and proliferation by regulating ribosome biogenesis, cell cycle progression, gene expression, metabolism, and physiology at the DNA, RNA and protein levels.
- RioK1 acts in response to nutritional availability, and possibly also to DNA damage, heat, osmotic, and oxidative stresses.
- RioK1 governs cellular health and survival by commanding p53 stability and activity via a complex signalling network that comprises myriad oncogenes.
- Elevated levels of RioK1, as observed across the cancer spectrum, correlate with high tumour grade, cancer aggressiveness, and low patient survival.
- Elevated levels of RioK1 trigger p53 degradation and cause resistance to radiotherapy, while depleting it stabilises p53 and sensitises cancer cells to treatment.

## Open questions

- Do cancer cells employ *RIOK1* overexpression as a strategy to inactivate p53?
- RioK1 has been studied minimally so far. Hence, what are its global targets and as-yet-unknown roles in cell biology that cause it to provoke tumorigenesis, invasion, and metastasis when dysregulated?
- Genetically depleting RioK1 in cell and mouse models led to the eradication of tumours driven by the oncogenes constituting the RioK1-p53 network. As a consequence, could drug-targeting RioK1 represent an efficient, broad-acting approach in cancer treatment?
- Drugs selectively inhibiting RioK1 do not exist. Given that RioK1 is a structurally atypical kinase/ATPase, can ligands be developed that target it with high specificity?

## Introduction

The tumour-suppressing transcription factor p53 promotes genetic stability, cellular health, and survival in response to myriad stresses. These include DNA damage, nutrient deprivation, hypoxia, heat shock, ribosome depletion, endoplasmic reticulum stress, viral infection, and oncogenic activation [1]. Since its discovery in 1979, numerous proteins and regulatory RNAs have been shown to promote or antagonise p53 expression, stability, and activity [1]. These include the transcription factors c-Myc and NF-κB, kinases Akt, PI3K, mTOR, Aurora A and B, the GTPases H/N/K-Ras, G3BP Stress Granule Assembly Factor 2 (G3BP2), E3 ubiquitin ligase MDM2, E3 SUMOylase TRIML2, long non-coding RNAs MILIP and LincROR, as well as microRNA miR-204-5p. How their activities combine into a coherent signalling circuit that receives the above-mentioned, dissimilar cues, to then relay them to p53 remains unclear.

**Fig. 1:**
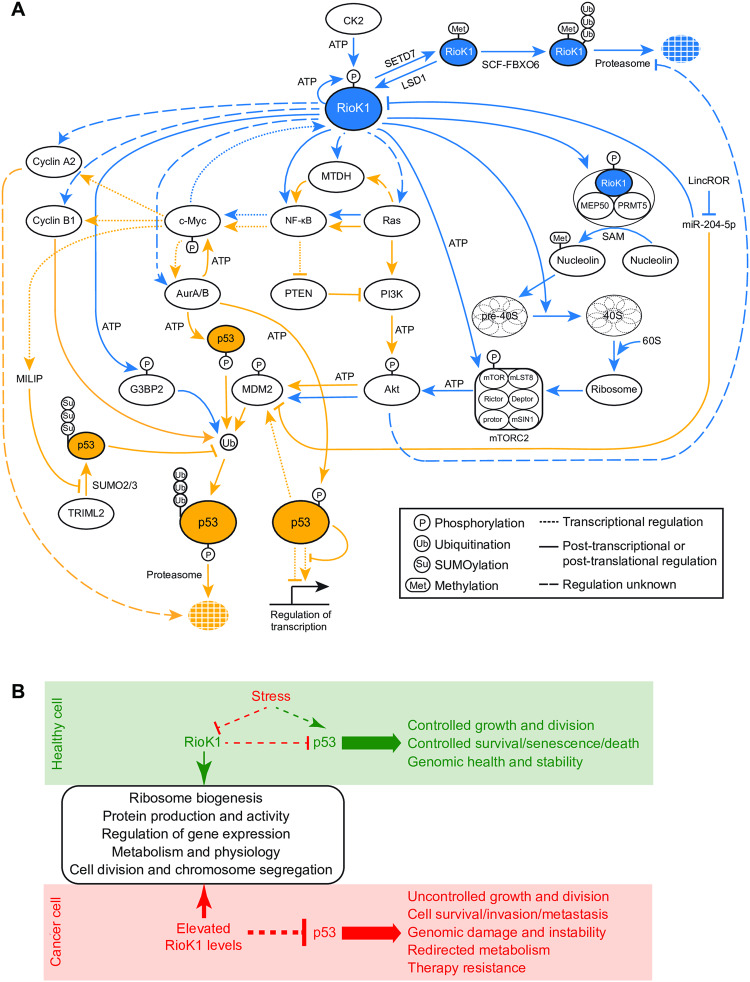
RioK1 and its downstream network regulate p53 activity. **A** The activity of kinase/ATPase RioK1 is regulated by methylation, phosphorylation, and ubiquitination, via lysine N-methyltransferase SETD7, lysine-specific demethylase LSD1, casein kinase CK2, and the E3 ubiquitin ligase SCF complex comprising subunit FBXO6, respectively. As indicated by the blue lines, RioK1 promotes pre-40S small ribosomal subunit maturation, both by releasing biogenesis factors, and by being part -as an adaptor- of the protein arginine methyltransferase 5 complex (PRMT5, also known as the methylosome) that activates nucleolin, which regulates pre-rRNA production and processing. RioK1 also turns on the mTORC2 kinase complex by targeting kinase mTOR. Next, mTORC2 activates protein kinase Akt, which triggers the E3 ubiquitin ligase MDM2 complex into poly-ubiquitinating p53, licensing its degradation by the proteasome. RioK1 similarly promotes Akt activity via a signalling cascade comprising GTPases K/H/N-Ras and protein kinase PI3K. Either directly or via the Ras GTPases, RioK1 supports metadherin (MTDH) expression, which in turn can inhibit p53 via the NF-κB/PTEN/PI3K/Akt pathway. Transcription factor c-Myc drives RioK1 expression and is in turn regulated by NF-ĸB via RioK1 or RioK1-Ras. c-Myc also activates the expression of kinases Aurora A and B, the latter of which phosphorylates and stabilises c-Myc. Both Aurora kinases can phosphorylate p53 at different residues, which can reduce p53 transcription activity or stimulate p53 poly-ubiquitination by MDM2, followed by proteasomal degradation. c-Myc also activates the expression of the long non-coding RNA MILIP, which, by competing for binding to p53, inhibits the SUMOylation of p53 by TRIML2. The latter safeguards p53 by preventing its poly-ubiquitination. Long non-coding RNA LincROR, acting as a sponge, captures and impedes miR-204-5p activity, which negatively regulates RioK1 and MDM2. c-Myc further promotes the expression of cyclins A2 and B1, which provoke p53 degradation. Finally, RioK1 downregulates p53 by directly activating G3BP2, which stimulates p53 ubiquitination by MDM2. The orange lines indicate the events that regulate p53 stability, activity, or turnover. **B** Schematic summary of the processes that RioK1 regulates (rounded rectangle). In healthy cells, the RioK1-p53 network promotes growth and viability; survival, senescence, and death, and ensures genomic health and stability. Under stressful conditions, RioK1 and its activities are downregulated, while p53 is activated. Overexpression of RioK1, as observed with high frequency in cancers (Fig. 2A, C) results in p53 degradation. The latter causes genetic instability, dysregulation of growth and proliferation, tumorigenesis, invasion and metastasis, and resistance to therapy. For details, see the main text.

A recent study by Chen et al. [2] revealed that kinase/ATPase RioK1 promotes G3BP2 phosphorylation. This event instigates p53 ubiquitination and degradation, and provokes radiotherapy resistance. This observation led us to probe further whether RioK1 could be more extensively involved in p53 regulation. Indeed, a substantial number of proteins and processes that determine p53 activity depend on RioK1 for their expression, activity, and stability. As a result, we here present the RioK1-p53 response network (Fig. 1A) comprising RioK1-controlled events (blue lines) that signal p53 (orange lines). Some of the latter regulators in turn influence RioK1 expression/activity/stability through feedback loops. *RIOK1*, the p53-encoding gene *TP53*, and the protein “nodes” that lie at the core of the RioK1-p53 network are found with high frequencies to be amplified, overexpressed, or mutated across the cancer spectrum [3, 4]. The consequent dysregulation of the RioK1-p53 response network may culminate in p53 inactivity, tumorigenesis, and ineffective treatment.

## RioK1: a multipurpose kinase and ATPase

RioK1 belongs to the RIO family of atypical protein kinases/ATPases that further include RioK2, RioK3, and RioB, which evolved from Archaea to humans [5]. While RioK1 is the best-studied member, its known activities (Fig. 1A, B; Table 1) remain limited. RioK1 is most noted for its participation in ribosome biogenesis. As part of the PRMT5 complex (also known as the methylosome), RioK1 promotes the di-methylation of nucleolin, which then stimulates rDNA transcription, pre-rRNA maturation, rRNA folding, and ribosome assembly [6]. As an ATPase, RioK1 supports the release of biogenesis factors during pre-40S small ribosomal subunit maturation [7–9]. Aside from these contributions, RioK1 also phospho-activates protein kinase Akt [10–12]. Together with RioK2, RioK1 turns on kinase mTOR, which, as part of the mTORC2 complex, phosphorylates Akt [13]. Hence, knocking down RioK1 disrupts both ribosome production and Akt signalling, which triggers the ribosomal-stress checkpoint [14]. The latter activates p53 (Fig. 1A), which halts the cell cycle to then eliminate the arrested cell through apoptosis [13].

**Table 1:** RioK1 targets and activities that culminate in p53 degradation when RioK1 levels are elevated.

Pathway	Target	Activity	References
Cell growth and proliferation	Pre-40S small ribosomal subunit	As an ATPase, RioK1 supports ribosome production by releasing biogenesis factors during pre-40S small ribosomal subunit maturation.	[7, 8]
	Nucleolin	RioK1 is part of the methylosome PRMT5-MEP50 complex that di-methylates nucleolin, which promotes rDNA transcription, pre-rRNA maturation, and rRNA folding.	[6, 67**]**
	c-Myc	RioK1 supports c-Myc protein translation; c-Myc in turn activates RioK1 transcription (positive feedback loop).	[19–21]
	mTORC2	RioK1 and RioK2 activate mTORC2 via phosphorylation of protein kinase subunit mTOR.	[13]
	Akt	RioK1 promotes Akt phosphorylation via mTORC2 and Ras/PI3K signalling pathways.	[10–13]
	Aurora kinases A and B	RioK1 supports the expression of Aurora kinases A and B via c-Myc.	[15, 31–33]
	Cyclin B1	RioK1 promotes cyclin B1 translation via c-Myc/WDR4 signalling.	[11, 15, 35]
	Cyclin A2	RioK1 promotes cyclin A2 transcription via c-Myc.	[15, 36]
	Metadherin	RioK1 supports metadherin expression. The latter activates the NF-ĸB and PI3K/Akt signalling pathways.	[15, 18, 47, 48]
Cell survival	H/N/K-Ras	Cell-based RNAi screens identified RioK1 as being required for Ras-driven cancer cell survival.	[13, 61]
	NF-ĸB	RioK1 activates NF-ĸB signalling via the Ras pathway.	[15]
	G3BP2	RioK1 directly phosphorylates G3BP2 to promote p53 ubiquitination by MDM2.	[2]
Epithelial-mesenchymal transition (EMT)	E and N-cadherin, vimentin, STAT3 and TWIST1	RioK1 activates the EMT via c-Myc, NF-ĸB and Akt signalling routes that promote the expression/activation of the STAT3 and TWIST1 transcription factors. These induce the expression of N-cadherin and vimentin, and downregulate E-cadherin production.	[13, 15, 16, 42–44]

Depleting RioK1 also affects the phosphorylation and protein levels of key cell cycle regulators, including cyclins A2 and B1, kinases Aurora A and B, ribonucleoprotein LARP1, and the microtubule-destabilising protein stathmin-1 [15]. These observations underscore the importance of RioK1 in controlling cellular proliferation. *RIOK1* overexpression promotes cell migration and invasiveness via the epithelial-mesenchymal transition (EMT) pathway. Knocking down RioK1 annihilated EMT activity due to increased E-cadherin levels, decreased concentrations of N-cadherin, vimentin, and matrix metalloproteinase-2, caused by the downregulation of transcription factors STAT3 and TWIST1 [2, 11, 15, 16]. Under the same conditions, oncogenic protein metadherin (MTDH), which promotes Akt- and NF-κB−mediated signalling and metastasis [17, 18], is reduced in expression [15] (Fig. 1A).

The proto-oncogenic transcription factor c-Myc, together with transactivator MAPJD, drive *RIOK1* expression [19], while miR-204-5p antagonises c-Myc by downregulating the *RIOK1* transcripts [10] (Fig. 1A). RioK1, in turn, promotes the translation of c-Myc mRNAs [20], hence establishing a feedback loop that incites the transformation and metastatic capacity of c-Myc. Since c-Myc and RioK1 partake in the same processes (including gene expression, ribosome biogenesis, cell cycle control, metabolism, motility, and invasion), RioK1 might well be a key downstream facilitator of c-Myc−mediated tumorigenesis. *RIOK1* expression is also stimulated by E2F transcription factors [21]. Its promoter further comprises a CpG island, and its chromatin is enriched with transcription-activating H3K4me3 marks [21]. ChIP-Seq and ChIP-chip experiments have revealed the presence at the *RIOK1* promoter of the oncogenic transcription factor FOXM1, and of the tumour-suppressing lysine-specific histone demethylase 6 A (KDM6A), respectively [22]. Additional research is needed to decipher *RIOK1* regulation at the expression level, which appears much more complicated than currently appreciated.

RioK1 levels and activity are also controlled post-translationally (Fig. 1A). The lysine N-methyltransferase SETD7 methylates RioK1 at K411 to facilitate its interaction with FBXO6; subunit of the E3 ubiquitin ligase complex SCF. The latter ubiquitinates RioK1 to trigger its proteasomal degradation. In contrast, casein kinase 2 (CK2) phosphorylates RioK1 at T410 to prevent K411 methylation, while lysine-specific demethylase 1 (LSD1) reverses RioK1 methylation by SETD7 [9, 16**]** (Fig. 1A). CK2 phosphorylates RioK1 in vitro also at S21 and S22 [23]. RioK1 phosphorylates itself at S407 [23], possibly to prevent its oligomerisation and maintain its most active, monomeric form [24]. Importantly, an inverse expression pattern between *RIOK1* and *SETD7*, and a positively correlating expression between *RIOK1* and *LSD1* or *CK2* have been observed in colorectal cancer, further substantiating the upregulation of RioK1 in a malignant context [16].

The biological activities of the proteins/substrates co-purifying with RioK1 [25] suggest roles of this enzyme beyond those known to date. These involve stress responses, metabolism, ribosome translation activity, protein turnover, chromatin remodelling and regulation of transcription, RNA processing and turnover, kinetochore assembly and activity. Proteins interacting with orthologue Rio1 in *Saccharomyces cerevisiae* mediate analogous functions [25, 26], suggesting the conservancy of many as-yet unexplored roles of RioK1/Rio1. For example, a recent study from our lab demonstrated that Rio1 and RioK1 downregulate centromere transcript levels to ensure the timely formation of structurally fit kinetochores, which promote faithful chromosome transmission during cell division [26].

## RioK1-regulated events affect p53 stability and activity

**Fig. 2:**
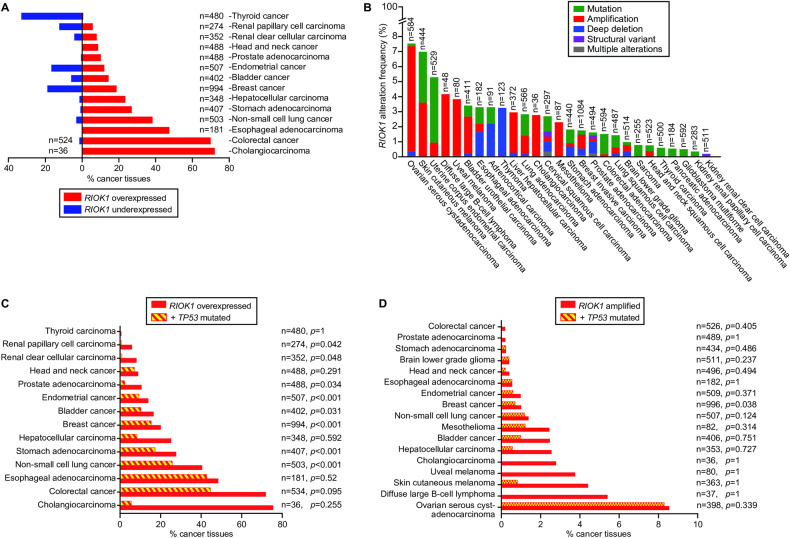
Frequencies of RIOK1 misexpression, amplification or alterations across the cancer spectrum. **A** Percentage of cancers that misexpress *RIOK1*. *n* = number of cases. **B** Percentage of cancers carrying specific alterations in *RIOK1*. *n* = number of cases. **C** Percentage of cancers that only overexpress *RIOK1* and of those that also carry mutations in *TP53*. *n* = number of cases, *p*-values indicate the statistical likelihood of *RIOK1* overexpression and *TP53* mutations coinciding in the same cancer. **D** Percentage of cancers with amplified *RIOK1* and of those that also carry mutations in *TP53*. *n* = number of cases, *p*-values indicate the statistical likelihood of *RIOK1* amplification and *TP53* mutations coinciding in the same cancer. The data in panels **A**, **B**, **C**, and **D** were obtained from the cBioPortal for Cancer Genomics v5.4.3 [3].

As noted earlier, cells depleted of RioK1 trigger the ribosomal-stress checkpoint [13], which signals p53 to halt cell division and induce apoptosis [13]. Just recently, RioK1 was shown to phosphorylate protein G3BP2 at T226, which provokes p53 ubiquitination and degradation [2] (Fig. 1A). Both findings highlight the important relationship between RioK1 and p53. A closer look at the presently known RioK1 protein targets (blue lines in Fig. 1A) and the proteins and RNAs that regulate p53 (orange lines in Fig. 1A) allowed us to unite them into a coherent signalling network. Given the roles of the proteins involved, this multi-functional network emerges as a manager of stress responses, ribosome production and activity, metabolism, cell growth and division, apoptosis, tumorigenesis, migration and invasion (summarised in Fig. 1B). Alterations in RioK1 activity due to overexpression, gene amplification or enhanced stability (Fig. 1A and Fig. 2A, B) could serve to inactivate p53, as similarly caused by *MDM2* overexpression, resulting in unrestrained growth and proliferation, genetic instability, increased survival, invasion, and metastasis, and ineffective cancer therapy (Fig. 1B).

Next to p53 carrying loss-of-function mutations, wild-type p53 may turn oncogenic when dysregulated at the transcription or protein level (e.g., misexpression, mislocalisation to the cytoplasm precluding its nuclear activities, inactivation by viral oncogenic proteins binding to p53) [27]. A constitutive degradation of p53 is observed in cells with amplified or overexpressed *MDM2*, or that are incapable of inhibiting MDM2 [28]. As outlined in Fig. 1A, the RioK1-activated mTORC2 kinase complex and the Ras GTPase-PI3K axis downregulate p53 via kinase Akt, which stimulates MDM2 [29, 30]. Furthermore, c-Myc, with or without support from H/N/K-Ras and transcription factor NF-κB, drives the expression of Aurora A and B [31, 32]. While Aurora B can phosphorylate and stabilise c-Myc [33], both kinases also target p53 to either reduce its activity, or to stimulate its ubiquitination by MDM2 [31, 34] (Fig. 1A). Moreover, c-Myc activates the expression of methyltransferase WDR4, which catalyses the 7-methylguanosine modification at position 46 of certain tRNAs, therewith stimulating the translation of cyclin B1 transcripts. Cyclin B1 promotes p53 ubiquitination and degradation [35]. Cyclin A2, transcriptionally controlled by c-Myc [36], can also inhibit p53 and apoptosis [37, 38] through mechanisms that remain unclear. Cyclin A2 further triggers the EMT pathway via integrin signalling [39].

c-Myc promotes the production of long non-coding RNA MILIP [40]. By competing for binding to p53, MILIP prevents p53 SUMOylation by TRIML2, allowing for p53 to be ubiquitinated [40]. Finally, long non-coding RNA LincROR sequesters miR-204-5p, which downregulates RioK1 and MDM2 [41] (Fig. 1A). Notably, c-Myc, NF-κB [18], Ras, and PI3K/Akt [42] collaborate to induce the expression or activation of EMT transcription factors STAT3 [43] and TWIST1 [44], both of which can regulate p53. Indeed, by interacting with p53, TWIST1 prevents p53 phosphorylation, needed for its stability, resulting in p53 degradation [45]. STAT3 binding to the promoter of *TP53* represses its transcription [46]. Alongside, MTDH promotes PI3K/Akt activity directly or by acting on NF-κB [47], which in turn inhibits PTEN, causing p53 degradation [17, 48].

The upstream cues that RioK1 may transmit to p53 comprise nutrient availability. In humans and *Drosophila*, RioK1 activates the mTORC2 complex [13], which signals the presence of nutrients in order to adjust metabolism, protein synthesis, growth, and proliferation. In *Saccharomyces cerevisiae*, orthologue Rio1 restricts growth and division amid nutritional deprivation, while promoting both during nutrient abundance. Under the latter circumstance, Rio1 auto-activates itself at the transcription level [25]. Transcriptome, interactome, and kinome analyses have indicated that yeast Rio1 might also signal heat, osmotic, and oxidative stresses [25]. In Archaea, often isolated from extreme environments, *RIO1* expression and activity are upregulated in response to UV irradiation [49–51] and gamma-ray treatment [52], further suggesting a role of this conserved kinase/ATPase in the DNA-damage response. Probing these observations in human cells will be important to further consolidate the relationship between RioK1, p53, and cell survival.

## RioK1 as a broad-acting anti-cancer drug target

RioK1 is essential for (cancer) cell viability [21]. Indeed, only a low percentage (< 3%) of cancers carry a deep deletion or mutations in the gene (Fig. 2B), but grow and proliferate due to still unrecognised genetic adaptations. Pan-cancer studies [3, 4, 9] have also revealed that *RIOK1* is found amplified with low frequency (Fig. 2B, D), while its overexpression is prominent across the cancer spectrum (Fig. 2A, C). In contrast, only few malignancies suffer from *RIOK1* underexpression, with thyroid, prostate and breast cancers being the most prevalent (Fig. 2A). Elevated levels of RioK1 may derive from a transcriptional upregulation by c-Myc [21] and/or from increased protein stability via CK2 or LSD1 [16] (Fig. 1A). Increased concentrations of RioK1 correlate with high tumour grade, cancer aggressiveness, and low patient survival [10, 11, 15]. Upon *RIOK1* overexpression, p53 becomes degraded and cancer cells turn refractory to therapy [2] (Fig. 1B). Examples include the resistance of colorectal cancer cells to radiotherapy [2], of non-small cell lung cancer cells to cisplatin [11, 53], of oestrogen receptor-positive breast cancer cells to tamoxifen [54], and of colorectal cancer cells to 5-fluorouracil [55]. Malignancies in which p53 is underexpressed, dysregulated or mutationally inactivated similarly become resistant to therapy [56], reinforcing the hypothesis that the noted chemo- and radio-resistance of *RIOK1* overexpressing cancers could well be due to a loss of p53 activity. No more than an extremely low percentage of only breast cancers (<1% of 996 cases analysed) exhibited a significant co-occurrence of amplified *RIOK1* and mutations in *TP53* (Fig. 2D). In contrast, a high percentage of endometrial, bladder, breast, and non-small cell lung cancers (ranging from 10-26%, Fig. 2C) revealed a significant, positive correlation between the occurrence of *RIOK1* overexpression and presence of mutations in *TP53* [3], indicating that the latter condition is not mutually exclusive. This revelation warrants additional research as it may well advance our understanding of how elevated RioK1 levels/activity can affect (cancer) cell biology when p53 is mutated.

Downregulating *RIOK1* was shown to halt the proliferation and invasiveness of EGF-driven and Ras-addicted cancers [9, 10, 13]. Other studies demonstrated the lethality of *MTAP*-deleted [57–59] or Ras-driven malignancies [15, 60, 61] when RioK1 was depleted. These findings exposed the genetic vulnerability to RioK1 deficiency of cancers driven by K/H/N-Ras, and other oncogenic factors constituting the RioK1-p53 network (e.g., c-Myc, Akt [12], mTOR), suggesting that drug-targeting RioK1 may represent a successful strategy in the cancer clinic. During evolution, the catalytic domains of most eukaryotic protein kinases remained structurally similar or “typical”. However, the kinase/ATPase domain of RioK1 developed atypically [5] as its C-terminal lobe contains only three of the six canonical α-helices, while two additional α-helices lie adjacent to the five β-sheets, thereby extending the N-terminal lobe. RioK1 also comprises a flexible 31-residue insertion between αC and β3, and lacks the activation loop [8, 62, 63]. Its unique makeup could inspire the development of highly selective RioK1 inhibitors. Toyocamycin, identified 67 years ago as an anti-*Candida* antibiotic produced by *Streptomyces toyocaencis* [64], is currently used to inhibit RioK1 in lab settings [2, 21, 24]. Since this compound is an ATP analogue it might well inhibit other (a)typical protein kinases. Unfortunately, data in this regard are not available. In recent work, Levosimendan, a hydrazone and pyridazine derivative used to treat heart failure, was predicted computationally to target RioK1 in an ATP-competitive manner [65]. Indeed, many cancers proved sensitive to Levosimendan with the strongest anti-neoplastic effects being observed against hematopoietic lymphoma cell lines. However, this compound might also target other kinases, including RioK3 [65], but experimental evidence in this regard remains lacking. Recently, Nintedanib, a tyrosine kinase inhibitor approved for use in idiopathic pulmonary fibrosis, also targeted RioK1 in colorectal cancer cells [66]. More desirable precision approaches could involve developing ligands that target the post-translational modifications of RioK1 (p-S407, p-T410, met-K411), which determine its activity and stability. Alternatively, the unique folds that surround its catalytic domain or RioK1-substrate interactions could be probed as well. As for the latter, one recent study reported a macrocyclic compound that annihilated the RioK1-PRMT5 interaction in vitro [67], which in human cells, could provoke the ribosomal-stress response and p53-induced apoptosis. Small molecules that restore the activity of loss-of-function mutant p53 are also being developed [68]. These could be combined with compounds that disrupt the MDM2-p53 interaction [56] or that target RioK1 to enhance the stability of re-activated mutant p53, similar to inhibiting RioK1 in cancer cells expressing wild-type p53 [2]. Recent reports revealed that genetically downregulating or drug (Nintedanib) treating RioK1 abrogated the growth and proliferation of malignancies expressing gain-of-function mutant p53 [15, 66]. However, considering that neither p53 stability nor activity was probed, and given that RioK1 mediates myriad roles in cells, while Nintedanib does not target RioK1 exclusively, it cannot be excluded that secondary activities were aspecifically affected in both studies, causing the observed cytotoxicities.

Taken together, we wish that the signalling network outlined here will guide and advance future studies of RioK1, p53, and their functional relationships; establish RioK1 as a valuable biomarker, and inspire new therapies that target RioK1 in a broad range of cancers driven by mutated p53 or a dysregulated RioK1-p53 network.
